# In search of a daily physical activity “sweet spot”: Piloting a digital tracking intervention for people with multiple sclerosis

**DOI:** 10.1177/2055207619872077

**Published:** 2019-08-21

**Authors:** Emil Chiauzzi, Eric B Hekler, Jisoo Lee, Auriell Towner, Pronabesh DasMahapatra, Marcy Fitz-Randolph

**Affiliations:** 1 PatientsLikeMe, Inc., Cambridge, MA, USA; 2 School of Nutrition and Health Promotion, Arizona State University, Phoenix, AZ, USA

**Keywords:** Activity tracker, multiple sclerosis, self-experimentation, self-management, behavior change, patient-centered, pacing

## Abstract

**Objective:**

This pilot study tested a course-based intervention to help people with multiple sclerosis (MS) match their daily activity to symptom severity (“sweet spot”) using wearable activity trackers.

**Methods:**

This two-phase study recruited online research network members reporting MS and who were utilizing Fitbit One™ activity trackers. In the first phase, participant interviews assessed demand based on physical activity and the use of behavior-change techniques. The second phase assessed the demand, limited efficacy, acceptability, and practicality of a “Wearables 101” course that integrated behavior change and self-experimentation principles. Tracker data were used to determine the percent of matches between daily symptom-based step goals and step counts.

**Results:**

Participants expressed demand in the form of interest in gaining insights about a possible “sweet spot” behavioral target, if a system could be produced to support that. Limited efficacy results were mixed, with approximately one-third of participants dropping out and only half matching their daily target goals for at least 50% of days. In terms of practicality, participants commented on the burden of daily measurement and the need for a longer baseline period. Participants noted that tracking helped support an understanding of the link between activities and symptom severity, suggesting acceptability.

**Conclusions:**

Results suggested that the intervention demand and acceptability criteria were demonstrated more strongly than limited efficacy and practicality. The matching intervention tested in this study will require refinement in baseline measurement, goal definition, and reduced data-gathering burden. Such changes may improve efficacy and practicality requirements and, by extension, later impact of the intervention on MS outcomes. Overall, these results provide justification for additional work on refining the intervention to increase practicality and efficacy.

## Introduction

Technological progress has fostered the development of a variety of wearable devices for self-tracking. Physical activity monitors, with their ability to capture free-living walking behavior in the real-world environment, may provide insight into the progression and impact of illnesses.^1^ Studies have shown the usefulness of physical activity monitors in ostensibly healthy elderly or adult populations,^2^ as well as in cardiac post-surgical mobility recovery,^3^ diabetes,^4^ and multiple sclerosis (MS).^1^

With the advent of wearable tracking devices and the “quantified self” movement, many people are seeking to improve their wellness and medical conditions through self-tracking and self-experimentation with lifestyle interventions related to physical activity, sleep, and diet.^5,6^ Hekler and Burleson^7^ and Lee et al.^8^ have advanced the concept of a “self-experimentation toolkit,” which enables users to develop and individually test highly individualized behavior-change plans via positive reinforcement, stimulus control, self-reward, or other behavior-change techniques. They have developed this concept into a simple process that allows users to select a target behavior, personal goals related to that target behavior, cueing strategies, and self-rewards, and then evaluate if the strategies are helpful or not.^8^ An adaptive intervention framework acknowledges the value of context by utilizing personal daily assessments of symptoms and physical activity to determine health goals and behavior. This self-experimentation concept is applicable to wearable use and reflects the patient-centeredness necessary to engage users in continued use of these devices.^9^

MS may be a particularly good target for a self-experimentation intervention that focuses on walking because about half of those with relapsing–remitting MS will need walking assistance within 15 years of diagnosis,^10^ and people with MS are often physically inactive.^11^ Physical activity can lead to quality-of-life improvements in people with MS, and people with MS with mild to moderate disability are encouraged to perform at least 30 minutes of moderate-intensity physical activity twice a week.^12^ Exercise has been referred to as the “missing prescription” in MS.^13^

Building on the self-experimentation concept, we interviewed people with MS to explore demand in two phases. In Phase I, we interviewed people with MS who utilize and incorporate wearable devices and behavior-change principles into their daily activity. Based on this input, we then conducted Phase II to design and pilot test a “Wearables 101” course to help people with MS achieve daily step goals based on a personal “sweet spot” step goal target and their daily symptom status. A key consideration was how well participants could modulate their daily activity based on wearable device feedback. Basing our feasibility criteria on four of eight criteria delineated by Bowen et al.,^14^ we assessed demand (use of selected intervention activities in a defined population and stated interest in the concept), acceptability (how do participants react to the intervention), limited efficacy (the degree to which the intervention produced the intended results), and practicality (extent of delivery when resources or commitment are constrained).

## Phase I: Qualitative study and course development

### Methods

The objective of Phase I was to gather information about the use of a wearable activity tracker and associated behavioral strategies from participants reporting a diagnosed of MS. For both phases, participants were recruited through PatientsLikeMe (PLM), an online research platform that allows members to share personal health data. This sample was recruited from a group of 248 participants with MS who had completed a previous wearable tracker study in 2014.^15^ In this study we evaluated the feasibility of deploying activity monitoring devices, user acceptance and adherence to wearing devices, and data collection among device users. MS patients in this study reported an overall highly positive user experience, as well as finding the device a useful aid in quantifying their walking. Many of these participants continued to sync their data on the PLM platform after the study ended.

#### Participants

Members were invited to participate in Phase I interviews based on the following eligibility criteria: (1) participation in the previous MS wearables study, (2) permission for PLM to collect data from their Fitbit One™® wearable device, and (3) Fitbit One™ use for at least 1 month prior to beginning of the study.

The interviewee sample consisted of seven participants with MS (female = 6, male = 1) between 50 and 68 years of age. The length of time since diagnosis ranged from 9 to 37 years. The MS types reported were progressive relapsing (*n* = 1), secondary progressive (*n* = 2), relapsing–remitting (*n* = 2), and primary progressive (*n* = 2). In addition, the functional disability score as measured by the Multiple Sclerosis Rating Scale (MSRS)^16^ ranged from 7 (10^th^ percentile) to 46 (65^th^ percentile), indicating mild to moderate disability. All participants were ambulatory, thus able to measure steps using wearable devices. All participants wore their devices daily and checked their data at least weekly. See Table 1 for more details.

**Table 1.** Characteristics of Phase I interview participants.

Interview #	Age	Sex	MS Type^a^	MSRS Score^b^	Current Daily Steps^c^
1	62	F	RRMS	14	3000
2	61	F	PRMS	39	7000
3	54	F	RRMS	35	3000
4	68	F	PPMS	7	4000–5000
5	55	F	SPMS	35	up to 10,000
6	63	F	SPMS	46	3000–5000
7	50	M	PPMS	42	5000

^a^MS Type: RRMS (relapsing–remitting), PRMS (progressive relapsing), PPMS (primary progressive), SPMS (secondary progressive)

^b^MSRS (Multiple Sclerosis Rating Scale) Score: most recent score before interview was conducted

^c^Current Daily Steps: general estimates reported by the patient, based on long-term Fitbit usage

#### Procedure

Eligible participants were sent an email invitation to participate in structured 60-minute phone interviews conducted by a research assistant and a senior research team member. In the interest of time and because of the funding constraints of this pilot project, interviews were audio-recorded but not transcribed verbatim. The interviewer took primary notes during the interviews while a second researcher listened and took secondary notes that were later used to confirm wording and concepts.

The guide for the interviews was a collaborative and iterative effort of the research team. The interviews covered: (1) Activity Level, Ability, and Symptoms (pre-condition and current condition physical activity, current physical abilities to conduct physical activity, types of physical activity, symptoms as barriers to physical activity); (2) Impact of Activity (effect of physical activity on symptoms); (3) Types of Support (social support for physical activity); (4) Adaptations (alterations in frequency, conditions, and physical activity types based on disease status); (5) Fitbit use (frequency, use of features, learning from usage, social support, use of other apps); and (6) Behavioral Techniques (e.g. self-rewards, goal-setting, practice, etc.). This information was gathered in order to understand MS challenges as well as how people with MS adapt their physical activity so that they can manage their symptoms better.

#### Data analysis

The analysis of the structured interviews involved two steps. First, the audio recordings were converted into notes that could be translated into high-level, recurring themes based on the priori concepts listed above. Because this was formative research, coding was collaborative rather than individual and we did not conduct inter-rater reliability or formal bias assessment. The senior researcher reviewed all themes with the research assistant, who had listened to interviews and provided additional notes. Any discrepancies about concepts or notes were discussed and resolved with the research team. Second, the senior researcher and research assistant identified and compiled themes into a report intended to inform the development of the wearables course. These themes were then reviewed and finalized with the rest of the research team.

### Results

#### Feasibility criterion 1: Demand (use of selected intervention activities in a defined population)

##### Modulating activity and MS symptoms

All of the participants discussed ways in which they have shifted the types of activities they do to compensate for their post-diagnosis levels of ability. Participants reported involvement in sports activities (yoga, curling, swimming, horseback riding) as well as activities of daily living (household chores, stair walking, gardening). All participants gave examples of the overall impact of physical activity on their symptoms and daily functioning, including feeling less anxious, more relaxed, less stiffness, feeling motivated to do more around the house, better sleep, reduced stress, ability to stay mobile, and more energy. Overall, participants acknowledged that the physical activity was adding a positive benefit in some way and thus there was motivation to continue. All participants used their Fitbits at least intermittently, with their reported average counts varying widely based on perceived level of disability. Participants mentioned being aware of the 10,000 steps goal but understood that applied to the general population and not people with MS or mobility issues.

While they were active, some of the interviewees noted dissatisfaction with their current level of activity, especially when compared with previous favorite activities such as dancing and biking. All participants reported times when activities caused them to “overdo it” and would result in weak legs, increased frequency of falls, increased fatigue, and feeling “crappy” the next day. Participants identified certain symptoms as potential barriers to activities, including fatigue, difficulty with balance, problems with dexterity, spasticity, restless leg, pain, overheating, and tripping. Other barriers included unpredictable schedules and hot weather.

##### Behavior-change observations

Participants reported that they viewed exercise and physical activity based on fluctuations in MS symptoms, so any course should be adapted to dynamic personal limitations (or abilities). As one participant stated,

“… MS is not constant and so it fluctuates all day long so what happens if it's none in the morning but by noon it's increased.”

Because many people with MS can be at least partially dependent on family members and friends, social aspects of daily activity plans are critical. One male participant reported,

“My wife has been a caregiver extraordinaire and it wasn't right away—it took her some time to recognize limitations, but there are things that I simply won't be able to do and she understands when she will need to help …”

Finally, people with MS may not respond to the competitive aspects of activity devices, for example the challenge to accomplish more each day or compete with others’ step counts. All participants mentioned an avoidance of 10,000 steps as a daily goal, because of potential greater fatigue the following day.

Participants related specific concepts corresponding to behavior-change strategies for maintaining physical activity. Although the use of these strategies is not consciously defined as behavior change, participants reported use of what could be labeled goal-setting, stating intentions, self-rewards, social support, and problem-solving.^17^ More specifically, they utilized *planning* (adding days of rest after a taxing activity day or on/after a treatment day), *schedule shifting* (completing strenuous activities earlier or later in the day because of heat effects on MS symptoms), *environmental adaptations* (finding environmentally controlled settings such as malls and “big box” stores), and *pacing* (reducing activity intensity, slowing down, and not pushing beyond recognized indicators such as reduced range of motion). Based on these interviews, it is apparent that participants utilize a variety of behavioral strategies in the daily self-management of MS. One participant listed her key planning steps as:

“Just to prepare to sit for a long period of time, where to sit in relation to a bathroom. How far am I going to walk, are there steps involved, what will the weather be like?”

## Phase II: Wearables 101 for multiple sclerosis pilot study

The objective of Phase II was to develop and test a pilot intervention to help people with MS match their daily activity to their current symptomatology so that they do not over- or under-extend themselves in their participation in physical activity.

### Methods

#### Participants

**Figure 1.**
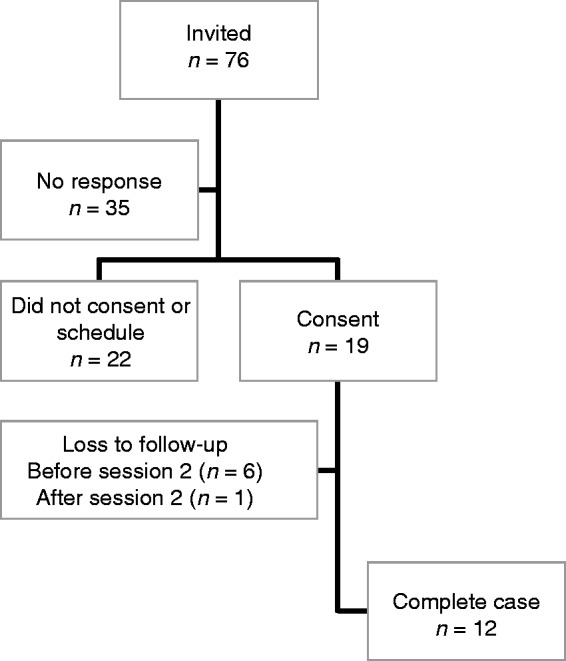
Flow of Participants through the Phase II Study.

Participants met the same criteria as in Phase I, and those who participated in the interviews in Phase I were allowed to participate in the Phase II course as well. Participants were not remunerated for their participation. Figure 1 describes the recruitment flow.

In total, 76 members met eligibility criteria and were contacted to participate in the wearables course; 41 participants responded to the invitations, 19 consented and scheduled a first session, and seven dropped out of the study (three after baseline survey, four after Session 1 completion).

Among the 12 participants who completed the course (female = 11, white = 10), the mean age was 54 years (ranged between 35 and 70 years). Six out of 12 participants were at least college graduates and eight participants reported at least one comorbid condition. At baseline MSRS scores ranged from 0 (1^st^ population percentile) to 60 (85^th^ population percentile) with a median of 39 (55^th^ population percentile) (see Table 2). Prior to our study, nine of 12 participants regularly used the Fitbit One™, with a median “use days/total days” = 44% (range 23% to 99%). Three participants had discontinued Fitbit use but started using Fitbit again to participate in the study.

**Table 2.** Baseline characteristics of Phase II study participants (N = 12).

Age (years), Mean ± SD	54 ± 10 (range 35–70)
Gender – female, *N* (%)	11 (92%)
Race – white, *N* (%)	10 (83%)
Education, *N* (%)	
Any college	6 (50%)
College graduate	4 (33%)
Advanced degree	2 (17%)
Reported comorbidities, *N* (%)	
None	4 (33%)
1	3 (25%)
2+	5 (42%)
MSRS^a^, Median score (Population Percentile)	39 (55^th^ percentile) Score range: 0 to 60
MET-min/wk^b^, Median score	1111 (range 0–7919; only 10 reports)
MS type	
Relapsing–Remitting	9 (75%)
Primary Progressive	1 (8%)
Secondary Progressive	2 (17%)

^a^: MSRS: Multiple Sclerosis Rating Scale; Population percentile from PLM database.

^b^: METs are multiples of the resting metabolic rate measured as MET-minutes/week score from the International Physical Activity Questionnaire (IPAQ).

#### Wearables 101 course development and delivery

We designed the pilot intervention to follow particular participant observations and suggestions in Phase I: (1) setting physical activity goals needs to be adapted each day; (2) any behavior-change plan must account for a myriad of fluctuating symptoms; (3) goals should be relative rather than based on a 10,000 step goal, accounting for lower than “average” and “peak” days across a predetermined period; (4) patients may ambulate using assistive devices and this needs to be accounted for in change plans; (5) we need to take note that patients utilize a range of activities that may not be amenable to Fitbit measurement, e.g. swimming and yoga; (6) focusing on steps is useful because participants tended not to note relationships among different Fitbit metrics (calories, distance, etc.) but do note relationships with between measured step levels and MS management; (7) participants are trying to perform against internal rather than external standards; (8) make the course more about behavior change than how to use Fitbits.

**Figure 2.**
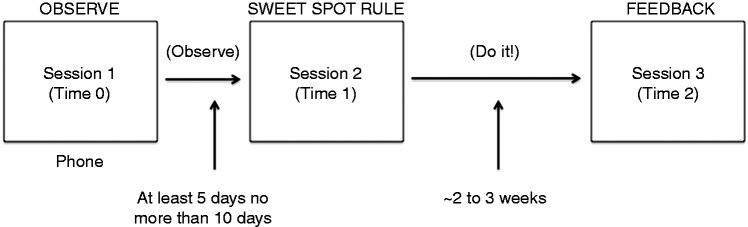
Flow of Wearables 101 Course.

To implement these goals, the Wearables 101 course was designed by coauthors (EH, JL) based on behavior change and adaptive intervention principles. By adopting a self-experimentation framework,^7,8^ participants were encouraged to engage in self-observation, rule-based goal-setting, and behavioral adaptations in physical activity based on the first two components. The resulting course was designed as a PowerPoint that walked participants through three, 30–60-minute phone sessions to set daily walking goals, track physical progress, and provide final results and course feedback (see Figure 2). A research assistant was trained by the investigators to review the tasks for each session in the PowerPoints, monitor adherence, respond to questions, and gather qualitative data through Session 3 feedback. The research assistant shared the PowerPoint with the participant and walked through the tasks and information to be gathered in each session.

Following the completion of two baseline measures (described below)—the International Physical Activity Questionnaire-Short Form (IPAQ-SF) and MSRS—participants were scheduled for a first session (“Observe”). The Session 1 phone call oriented participants to the course and explained the goals of the first week. Each participant learned about the concept of a “sweet spot” (matching activity to daily physical condition). Participants were asked to select all factors (e.g. pain, fatigue, concentration) that might affect their physical activity, and to rate them on a scale (None–Mild–Moderate–Severe) in the morning at the PatientsLikeMe website. Participants also completed a daily “Instant Me” global rating (*How are you feeling now?* 1 = very good, 5 = very bad) at the PatientsLikeMe website once in the morning and once in the evening. These ratings would be used to determine if the number of daily steps was well matched to daily functioning. Finally, participants were instructed to make sure their Fitbit was on so that their data could be synced with the PatientsLikeMe website.

After the observation period, the Session 2 “sweet spot rule” call was scheduled to check-in and review the data that had been collected by the patient. The multiple factors/symptoms they were tracking were narrowed down to one based on the data collected as well as their perception of what was the most impactful symptom on their steps and ability to stay active during the day. Participants were to: (1) rate their InstantMe and symptom in the morning; (2) set their step goal based on their predetermined rule; and (3) at the end of the day rate their InstantMe again noting if they met their goal and if they felt the goal helped them to get closer to their sweet spot. By relating their symptom ratings to their activity level (steps), participants could develop a subjectively driven strategy (“rule”) to define how many steps they could take on “good” vs. “bad” days. This rule would be used to help determine their daily step goal. For example, one participant set daily targets based on levels of pain. On days with no pain the goal was 6000 steps, mild pain reduced the step goal to 5000, and severe pain reduced the daily goal to 4000 steps. To help participants maximize their activity levels without overextending themselves, participants were instructed in integrating an adaptive behavioral technique. For example, one participant set a rule that on days when she did not overextend herself and had “steps left,” she could go out at night. As in Session 1, participants were asked to continue to use and sync their Fitbit. This activity occurred over the next 2–3 weeks. Rather than rely on a Fitbit default goal (e.g. 10,000 steps), participants focused on their own “sweet spot” step goal. Phone support with a research assistant was available if participants had tracking or course questions.

The final session, Session 3 (“Feedback”), involved reviewing the data that had been collected since Session 2. This involved understanding the patient’s perception of what they felt they learned the past 2 weeks, how the rule impacted them on a daily basis, if they felt they found their sweet spot, and then a brief discussion related to their overall experience with the course (what worked and did not work).

#### Measures

We covered the following topics within semi-structured interviews to probe for demand, acceptability, and practicality: challenges in sweet spot assessment and application, barriers and facilitators of behavior-change plans, issues regarding providing and managing personal data, observations about links between physical activity and symptoms, suggested changes for future behavior-change programs.

Three measures were used in this pilot test for the quantitative analyses to examine limited efficacy. Physical activity was measured as daily step count from the Fitbit One™ wearable device. Self-reported physical activity was measured by the IPAQ-SF,^18^ a self-administered 7-item scale that is designed to assess an individual’s health and level of physical activity. The IPAQ-SF estimates metabolic equivalents (METs), which are multiples of the resting metabolic rate measured as MET-minutes/week. The IPAQ-SF was administered before and after the course. MS severity was measured using the MSRS.^16^ The MSRS is a 7-item scale that measures MS severity (walking, upper limb function, vision, speech, swallowing, cognition, and sensation). Participants were asked to complete the MSRS before and after the course.

#### Data analysis

We analyzed qualitative data to assess demand, acceptability, and practicality. To identify interview themes, a research scientist and research assistant used a combination of deductive and inductive coding based on thematic analysis.^19^ All notes were collected and grouped by major areas of interest to the team a priori (use of devices, knowledge of how to use them, ideas for getting others to use them) as well as interview-derived themes based on participant perspectives (concerns about overactivity, variability of symptoms and disease course, effectiveness of step counts as a proxy for all kinds of activities). In addition, key quotes related to participants’ experience of the course were identified. As in Phase I, coding was collaborative and we did not conduct inter-rater reliability or formal bias assessment.

Quantitative data were analyzed to assess efficacy. Quantitative data were analyzed for all complete cases in the following steps: (1) descriptive statistics were computed or demographic variables; (2) measured individual daily activities (steps) over the course of the study were summarized descriptively (mean, median, coefficient of variation) including the overall match rate (number of days goals met/total number of course days); (3) data computed in step 2 were used to compute aggregate level descriptive statistics (before and after the course) and match rate (after the course).

### Results

#### Feasibility Criteria: Demand (use of selected intervention activities in a defined population) and acceptability (reactions to the use of an intervention like this in their daily lives)

The feedback regarding patient experience in the course was positive and suggested demand from patients for a tool that could support them in defining the sweet spot. The sweet spot concept appeared to have much intuitive appeal, but targeting daily activity using a morning rating was challenging for some participants. In addition, there was a carryover effect between days, as some participants required rest days after overly active days. Among the 12 participants who completed the pilot study, there were consistent (but not always successful) efforts to wear their devices, track their step counts, and manage their personal rules. Most referred to their daily efforts using “sweet spot” terminology and looking to make connections between their behavior and daily experience. For example, one participant stated,

“… we've been doing one mile around the block and I (am) tempted to maybe do a second mile and I think that my sweet spot is one mile because the second one starts making me feel shaky …” [P2]

There was general acceptability for a tool like this, if it could be implemented more effectively (see Feasibility Criterion 4). Several participants reported that the course helped them learn more about their bodies and more accurately determine daily activity levels, look for trends, and maintain motivation. The overarching key to the tracking was that it helped support personal understanding of the link between physical activity levels and symptom severity, for example, restless leg, foot drop, heat tolerance, pain, and balance problems. Participants also reported that they learned about pacing, activity types that help symptoms, and motivational factors. One participant noted,

“… the wearing of the wearable was not a problem at all and it made me be tuned in to the fact that when I woke up in the morning I should take stock of how I feel and pay attention to that and I like that and it made me be more aware I think …” [P18]

Another stated,

“I get spasticity and was noticing when I was running around that the spasticity in the afternoon gets worse in the morning than in the afternoon—I noticed that the spasticity was worse in the afternoon but it's also irritating because that's the time that I have available to me—happens more between 5 and 7 which is annoying and I can't change that.” [P13]

#### Feasibility criterion 3: Limited efficacy (the degree to which the intervention produced the intended results)

Results on the drop-out rates indicate that this type of tool could feasibly be utilized by some patients with MS, though not by all individuals. As an indicator of efficacy, 7/19 original participants dropped out (see Figure 1). Reasons for discontinuing course participation were: (1) not experiencing any MS symptoms, (2) losing or misplacing Fitbit, (3) did not respond to outreach, (4) worsening health issues, and (5) did not like using Fitbit.

In terms of limited efficacy results, the mean of daily step counts of participants over the course of the study ranged from 1478 to 13,300; the aggregate (mean of participant means) was 5397 (Session 1: 5291; Session 2 course onwards: 5567) and the mean match rate (% of goal match days) for participants ranged from 10% to 86% (mean 49%). Half of the participants achieved their daily target goals for at least 50% days, and only two matched their goals for more than 75% days. Dispersion from the mean activity level assessed by coefficient of variation was also variable across participants, ranging from 13% to 68% (Table 3). Overall, results suggest that the protocol as designed did not achieve acceptable match rates for most participants.

**Table 3.** Level of pre-post activity and match rate (N = 12).

Participant	Before course			After course				
	Days	Mean	CV^a^	Days	Mean	CV^a^	Match^b^	Match Rate^c^
1	8	3111	26	13	3589	47	9/13	69%
2	14	6619	14	14	6978	12	12/14	86%
3	7	7269	56	13	5117	33	3/12	25%
4	12	802	102	15	2019	40	4/13	31%
5	14	9981	50	14	14,625	15	9/14	64%
6	8	2698	38	13	2694	48	1/10	10%
7	14	4448	21	14	5400	32	4/9	44%
8	13	4039	33	14	3290	26	9/14	64%
9	11	4371	34	20	4163	62	2/13	15%
10	10	1949	34	14	2225	29	9/14	64%
11	10	13621	24	14	13063	31	5/6	83%
12	10	4580	26	13	3644	25	3/11	27%
Aggregate statistics^d^		5291	38		5567	33	.	49%
(Mean of participant means)								Range 10% to 86%

^a^ CV (Coefficient of variation) = Standard deviation (SD)/mean a measure of variability in relation to the mean.

^b^ Match shows concordance between daily goals with device measured activity within a ± 20% range. Data presented show total match days (numerator)/total course days (denominator). Note: Total course days may not equal total days in session 2 because of skipped course days.

^c^ Match rate is the percentage of days in which the daily step target was achieved during the course.

^d^ Aggregate statistics reflect the mean of the variables for all 12 participants.

As an illustration, participant 2 represents a high degree of adherence to the protocol (minimal dispersion for the mean, high matching to target goals). It should be noted that given the small sample of participants, outcome measures such as MSRS and MET-minimum/week were used for descriptive purposes and changes in pre-post scores were not analyzed. Based on these results, the tool was useful for some but not all individuals, thus suggesting limited efficacy. Additional refinement of the tool will be required to increase the total number of individuals who could benefit from it.

#### Feasibility criterion 4: Practicality (extent of delivery when resources constrained)

Participants noted several deficiencies that affected the practicality of the pilot course. First, the “low tech” nature of course delivery (PowerPoint), tracking, and logging (InstantMe ratings at the PatientsLikeMe website) was very burdensome. Throughout the course, participants stated a desire for a format that offered a more user-friendly data-capture method such as using mobile tracking and unifying data capture in one place. Second, it became clear that any technology-based method for adapting daily physical activity must deal more with the frequent fluctuations of chronic disease, for example, health status changes (simply having a bad day, having a flare up, difficulties with cognition, etc.), issues with tracker connectivity, or developing a cadence for the tracking portion of the course. Finally, some participants commented on the importance of an extended baseline period because of skewing effects of atypical activity weeks.

## Discussion

The purpose of this study was to examine how people with MS utilize and incorporate wearable devices and behavior-change techniques into their daily activity and how well they could modulate their daily activity based on feedback from wearable devices. To evaluate this, our primary focus was on assessing issues of feasibility^15^ including demand (interest in the use of selected intervention activities in a defined population), acceptability (reactions to intervention as implemented), limited efficacy (the degree to which the intervention resulted in the desired outcome of individuals hitting their “sweet spot” daily target), and practicality (extent of delivery when resources or commitment are constrained).

Results from our interviews indicated demand for the targeted type of support that would help them to define their “sweet spot” daily behavioral target, which was further reinforced in the pilot testing of the intervention. Results also indicated general acceptability of the intervention in the pilot study, as participants who set daily targets generally accepted and applied the “sweet spot” concept. In terms of limited efficacy, a large portion of individuals dropped out of use of the intervention, which is indicative of potential challenges for utilization of the current tool by some patients with MS. This dropout may also reflect the common experience that people abandon these devices as a result of data concerns, lack of need, or changes in circumstances or routines.^20^ Similar results were also found with regard to limited efficacy claims as not all individuals could achieve the desired sweet spot targets, though a strong sub-sample of participants did. Finally, in terms of practicality, results indicated that the current tool was not particularly practical as designed for most participants, as it was too burdensome and, simultaneously, not dynamic enough to be responsive to the needs of the patients.

The present pilot study contributes several key considerations in fitness tracker use and research with people with MS. First, most wearable tracker research focuses on technical or device-related challenges rather than exploring the potential barriers faced by users of physical activity tracking devices.^21,22^ Physical activity patterns are likely to be highly variable, vulnerable to environmental conditions, and may be interrupted because of symptom flares. Accounting for these factors is critical in understanding the raw numbers that are generated by these trackers.

Second, certain features that are considered desirable in a fitness framework may be less desirable in a chronic illness framework, for example, social comparison or competition features. Some studies have found that social functionality and competition to be motivators for maintaining engagement (e.g. Harrison et al.^9^), while others have found that many users find competitive features to be discouraging.^23^ The notion of sharing one’s daily steps output or competing with friends may be perceived as counterproductive when managing a chronic condition. Thus, user expectations in a wellness context may not apply in a disease-management context.^24^

Third, users tend to focus on the simplest data available and may not integrate that data into a personal or health context systematically. This may result from the greater accuracy of step counts compared with sleep data or energy expenditure in consumer activity monitors,^25^ and users may gravitate toward tracking their steps because other metrics (sleep, calories, etc.) are more difficult to track accurately. Among those experiencing chronic illness, consideration of other trackable metrics may provide greater insight into daily “sweet spots.”

Fourth, the use of wearables is complicated by within-day fluctuations in functioning related to MS. As a result, the oft-targeted 10,000 step daily goal may be unrealistic for many people with MS, because they report that their condition will likely worsen the next day. In this small sample, there was a high degree of variability in steps between and within participants across days in the study. Our limited data suggest that 10,000 daily steps may be excessive for people with MS, a finding that has been echoed in other physical activity studies with chronic diseases.^26^

### Study strengths and limitations

The strengths of this approach included a sample with a variety of MS types, the use of mixed methods, and a patient-centered approach to testing a program to match daily activity to physical capabilities in people with MS. However, there were several limitations to this work, including a small sample size of a relatively homogenous group of participants with online access and membership in an online community. The sample did not include those who were less engaged with technology. In addition, the sample was drawn from a larger group of people with MS who had been using wearables for almost a year, so may have represented the experiences of a more seasoned group of users of wearable devices. It may be that some individuals had settled into more ingrained use patterns. In terms of self-experimentation, this pilot study indicates that some individuals benefit from daily personal activity matching, and there was strong demand and acceptance of the self-experimentation and sweet spot concepts. However, the self-experimentation approach used in this study was likely not yet precise enough for most people with MS. Over one-third of participants dropped out of the study, thus suggesting efficacy challenges as the self-experimentation approach is currently designed. With that said, this drop-out rate is not unusual among wearable users, as one-third of US owners of wearable devices stop using these devices within 6 months of first use, typically because of problems in set-up, integration into lifestyles, and a lack of a clear value proposition.^27^ Future work is warranted on this concept to further improve the design of the intervention for real-world applications based on the more consistent results related to demand and acceptability and, among those that could implement the strategy in their lives, limited efficacy. In addition, successful matching requires longer baseline observation periods to stabilize individual activity patterns (mentioned by some participants), validation of matches with more than one daily rating (as symptom severity changes throughout the day), and repeated self-experimentation with behavior-change rules to refine stable, personalized “sweet spots.” Such strategies may also help stabilize daily activity goals to address the inevitable within-day fluctuations of MS symptoms.

## Conclusions

These results suggest further work should be invested in a more efficient delivery strategy to support MS patients in defining “sweet spot” rules for better understanding their personal “sweet spot” levels of physical activity. There is evidence for demand and acceptability, but only limited efficacy among those who could effectively use the self-experimentation protocol. Practicality is as yet unrealized, because there was inconsistent course usage even though it was implemented with a high degree of support and follow-up.
